# Supplementary material for: Classifying the non-metabolic demands of different physical activity types: The Physical Activity Demand (PAD) typology
Source: PLoS One. 2023 Oct 19;18(10):e0291782. doi: 10.1371/journal.pone.0291782 (PMC10586621; doi:10.1371/journal.pone.0291782)
Supplement: S1 Table — (DOCX) [file pone.0291782.s001.docx]

**S1 Table: Core 61 physical activity items**

| Physical Activities |  |
| --- | --- |
| Aerobics class | Fishing |
| Archery, non-hunting | Fitness class, aqua |
| Army type obstacle course exercise/boot camp training | Fitness class, resistance toning |
| Athletics | Football |
| Badminton | Frisbee |
| Basketball | Gardening |
| Bicycling, not stationary | Golf |
| Cleaning | Gymnastics |
| Cooking and food preparation | Handball |
| Cricket | Hockey, field and ice |
| Croquet | Home repair |
| Curling, bowls, bowling and shuffleboard | Home video/DVD workout |
| CV Exercise Machine e.g. treadmill, crosstrainer | Horseback riding |
| Dancing | Hunting |
| Diving | Man-powered boating |
| Exergaming e.g. Wii Sports | Martial arts/Combat sports |
| Figure skating and ice dancing | Orienteering |
| Pilates | Squash and racquetball |
| Playing children’s games | Surfing |
| Polo, on horseback | Swimming, laps |
| Resistance/strength Training | Synchronized swimming |
| Rock or mountain climbing | Table tennis |
| Rope skipping | Tai Chi |
| Rugby | Tennis |
| Running, not on treadmill | Trampolining |
| Skateboarding | Volleyball |
| Skating, ice, roller and in-line | Walking, not on treadmill |
| Skiing | Water polo |
| Skindiving and scubadiving | Windsurfing/sailing |
| Softball and rounders | Yoga |
| Spin/RPM/Cycle class |  |
