# Supplementary material for: Classifying the non-metabolic demands of different physical activity types: The Physical Activity Demand (PAD) typology
Source: PLoS One. 2023 Oct 19;18(10):e0291782. doi: 10.1371/journal.pone.0291782 (PMC10586621; doi:10.1371/journal.pone.0291782)
Supplement: S2 Table — (DOCX) [file pone.0291782.s002.docx]

**S2 Table: Results of cluster analysis 1 (mental demands of physical activity)**

| Mental demands | | |
| --- | --- | --- |
| Cluster 1 (Low demand) | Cluster 2 (Moderate demand) | Cluster 3 (High demand) |
| Aerobics Class | Archery, non-hunting | Badminton |
| Bicycling, not stationary | Army type obstacle course exercise/boot camp training | Basketball |
| Cleaning | Athletics | Football |
| CV Exercise Machine e.g. treadmill, crosstrainer | Cooking and food preparation | Handball |
| Exergaming e.g. Wii Sports | Cricket | Hockey, field and ice |
| Fishing | Croquet | Hunting |
| Fitness class, aqua | Curling, bowls, bowling and shuffleboard | Martial arts/Combat sports |
| Fitness class, resistance toning | Dancing | Polo, on horseback |
| Gardening | Diving | Rugby |
| Home video/DVD workout | Figure skating and ice dancing | Squash and racquetball |
| Resistance/strength Training | Golf | Surfing |
| Rope skipping | Gymnastics | Table tennis |
| Running, not on treadmill | Home repair | Tennis |
| Skateboarding | Horseback riding | Volleyball |
| Spin/RPM/Cycle class | Man-powered boating | Water polo |
| Swimming, laps | Orienteering | Windsurfing/sailing |
| Trampolining | Pilates |  |
| Walking, not on treadmill | Playing children’s games |  |
|  | Skating, ice, roller and in-line |  |
|  | Skiing |  |
|  | Skindiving and scubadiving |  |
|  | Softball and rounders |  |
|  | Synchronized swimming |  |
|  | Tai Chi |  |
|  | Yoga |  |
