# Supplementary material for: Classifying the non-metabolic demands of different physical activity types: The Physical Activity Demand (PAD) typology
Source: PLoS One. 2023 Oct 19;18(10):e0291782. doi: 10.1371/journal.pone.0291782 (PMC10586621; doi:10.1371/journal.pone.0291782)
Supplement: S3 Table — (DOCX) [file pone.0291782.s003.docx]

**S3 Table: Results of cluster analysis 2 (physical demands of physical activity)**

| Physical demands | | |
| --- | --- | --- |
| Cluster 1 (Low demand) | Cluster 2 (Moderate demand) | Cluster 3 (High demand) |
| Cleaning | Aerobics Class | Badminton |
| Cooking and food preparation | Archery, non-hunting | Basketball |
| Croquet | Army type obstacle course exercise/boot camp training | Cricket |
| Curling, bowls, bowling and shuffleboard | Athletics | Dancing |
| Fishing | Bicycling, not stationary | Diving |
| Gardening | CV Exercise Machine e.g. treadmill, crosstrainer | Figure skating and ice dancing |
| Home repair | Exergaming e.g. Wii Sports | Football |
| Hunting | Fitness class, aqua | Gymnastics |
| Spin/RPM/Cycle class | Fitness class, resistance toning | Handball |
| Walking, not on treadmill | Golf | Hockey, field and ice |
|  | Home video/DVD workout | Horseback riding |
|  | Man-powered boating | Martial arts/Combat sports |
|  | Orienteering | Polo, on horseback |
|  | Pilates | Rugby |
|  | Playing children’s games | Skateboarding |
|  | Resistance/strength Training | Skating, ice, roller and in-line |
|  | Rope skipping | Skiing |
|  | Running, not on treadmill | Softball and rounders |
|  | Skindiving and scubadiving | Squash and racquetball |
|  | Swimming, laps | Surfing |
|  | Tai Chi | Synchronized swimming |
|  | Yoga | Table tennis |
|  |  | Tennis |
|  |  | Trampolining |
|  |  | Volleyball |
|  |  | Water polo |
|  |  | Windsurfing/sailing |
