# Supplementary material for: Classifying the non-metabolic demands of different physical activity types: The Physical Activity Demand (PAD) typology
Source: PLoS One. 2023 Oct 19;18(10):e0291782. doi: 10.1371/journal.pone.0291782 (PMC10586621; doi:10.1371/journal.pone.0291782)
Supplement: S4 Table — (DOCX) [file pone.0291782.s004.docx]

**S4 Table: Results of cluster analysis 3 (social demands of physical activity)**

| Social demands | | |
| --- | --- | --- |
| Cluster 1 (alone) | Cluster 2 (alone or with others) | Cluster 3 (requires others) |
| Cleaning | Archery, non-hunting | Aerobics Class |
| CV Exercise Machine e.g. treadmill, crosstrainer | Army type obstacle course exercise/boot camp training | Badminton |
| Home video/DVD workout | Athletics | Basketball |
| Swimming, laps | Bicycling, not stationary | Cricket |
|  | Cooking and food preparation | Croquet |
|  | Dancing | Curling, bowls, bowling and shuffleboard |
|  | Diving | Fitness class, aqua |
|  | Exergaming e.g. Wii Sports | Fitness class, resistance toning |
|  | Figure skating and ice dancing | Football |
|  | Fishing | Handball |
|  | Gardening | Hockey, field and ice |
|  | Golf | Martial arts/Combat sports |
|  | Gymnastics | Playing children’s games |
|  | Home repair | Polo, on horseback |
|  | Horseback riding | Rugby |
|  | Hunting | Softball and rounders |
|  | Man-powered boating | Spin/RPM/Cycle class |
|  | Orienteering | Squash and racquetball |
|  | Pilates | Synchronized swimming |
|  | Resistance/strength Training | Table tennis |
|  | Rope skipping | Tennis |
|  | Running, not on treadmill | Volleyball |
|  | Skateboarding | Water polo |
|  | Skating, ice, roller and in-line |  |
|  | Skiing |  |
|  | Skindiving and scubadiving |  |
|  | Surfing |  |
|  | Tai Chi |  |
|  | Trampolining |  |
|  | Walking, not on treadmill |  |
|  | Windsurfing/sailing |  |
|  | Yoga |  |
