# Supplementary material for: Classifying the non-metabolic demands of different physical activity types: The Physical Activity Demand (PAD) typology
Source: PLoS One. 2023 Oct 19;18(10):e0291782. doi: 10.1371/journal.pone.0291782 (PMC10586621; doi:10.1371/journal.pone.0291782)
Supplement: S5 Table — (DOCX) [file pone.0291782.s005.docx]

**S5 Table: Physical activities categorized as low, moderate, or high multi-demand level**

| Low level | Moderate level | High level |
| --- | --- | --- |
| Cleaning | Aerobics | Badminton |
| Cooking and food preparation | Archery, non-hunting | Basketball |
| CV exercise machine e.g. treadmill, crosstrainer | Army type obstacle course/boot camp training | Cricket |
| Fishing | Athletics | Dancing |
| Gardening | Bicycling, not stationary | Diving |
| Home repair | Croquet | Figure skating and ice dancing |
| Home video/DVD workout | Curling, bowls, bowling, and shuffleboard | Football |
| Resistance/strength training | Exergaming, e.g. Wii Sports | Gymnastics |
| Running, not on a treadmill | Fitness class, aqua | Handball |
| Spin/RPM/Cycle Class | Fitness class, resistance/toning | Hockey, field and ice |
| Swimming, laps | Golf | Martial arts/combat sports |
| Walking, not on a treadmill | Horseback riding | Polo, on horseback |
|  | Hunting | Rugby |
|  | Man-powered boating | Skateboarding |
|  | Orienteering | Skiing |
|  | Pilates | Softball and rounders |
|  | Playing children’s games | Squash and racquetball |
|  | Rope skipping | Surfing |
|  | Skating, ice, roller and in-line | Synchronized swimming |
|  | Skindiving and scubadiving | Table tennis |
|  | Tai chi | Tennis |
|  | Trampolining | Volleyball |
|  | Yoga | Waterpolo |
|  |  | Windsurfing |
