# Supplementary material for: Classifying the non-metabolic demands of different physical activity types: The Physical Activity Demand (PAD) typology
Source: PLoS One. 2023 Oct 19;18(10):e0291782. doi: 10.1371/journal.pone.0291782 (PMC10586621; doi:10.1371/journal.pone.0291782)
Supplement: Attachment — Submitted filename: Response to Reviewers.docx [file pone.0291782.s008.docx]

Dear Editor and Reviewers,

Thank you for your constructive feedback on our manuscript PONE-D-23-21859 “Classifying the non-metabolic demands of different physical activity types: the Physical Activity Demand Typology”. We have addressed each point in turn and summarise the changes made below in red.

________________________________________________________________________________

(i) Editorial comment - If applicable, we recommend that you deposit your laboratory protocols in protocols.io to enhance the reproducibility of your results. Protocols.io assigns your protocol its own identifier (DOI) so that it can be cited independently in the future. For instructions see: [https://journals.plos.org/plosone/s/submission-guidelines#loc-laboratory-protocols](https://eur03.safelinks.protection.outlook.com/?url=https%3A%2F%2Fjournals.plos.org%2Fplosone%2Fs%2Fsubmission-guidelines%23loc-laboratory-protocols&data=05%7C01%7Cj.allan%40abdn.ac.uk%7C6055c768c26d4b4a856408db9371109d%7C8c2b19ad5f9c49d490773ec3cfc52b3f%7C0%7C0%7C638265886633825430%7CUnknown%7CTWFpbGZsb3d8eyJWIjoiMC4wLjAwMDAiLCJQIjoiV2luMzIiLCJBTiI6Ik1haWwiLCJXVCI6Mn0%3D%7C3000%7C%7C%7C&sdata=cn3%2Fwek%2BFkAQrfDosJDjl3mJUUBbB82pIcr0ZNrLdj8%3D&reserved=0).

We have deposited the study protocol in protocols.io as suggested and inserted the DOI (dx.doi.org/10.17504/protocols.io.yxmvm32kbl3p/v1) into the Materials and methods section of the paper.

1. Please ensure that your manuscript meets PLOS ONE's style requirements, including those for file naming. The PLOS ONE style templates can be found at 
<https://journals.plos.org/plosone/s/file?id=wjVg/PLOSOne_formatting_sample_main_body.pdf> and 
<https://journals.plos.org/plosone/s/file?id=ba62/PLOSOne_formatting_sample_title_authors_affiliations.pdf>

We have reviewed the manuscript style against PLOS ONE’s requirements and made small formatting changes (e.g. to level headings and the titles of supplementary information files) throughout to ensure alignment.

2. Thank you for stating the following financial disclosure: 
   "This work was funded as part of a PhD Studentship awarded to CR by the University of Aberdeen"

Please state what role the funders took in the study.  If the funders had no role, please state: "The funders had no role in study design, data collection and analysis, decision to publish, or preparation of the manuscript." Please include this amended Role of Funder statement in your cover letter; we will change the online submission form on your behalf.

We have amended the financial disclosure statement in the cover letter to read: "This work was funded as part of a PhD Studentship awarded to CR by the University of Aberdeen. The funders had no role in study design, data collection and analysis, decision to publish, or preparation of the manuscript." We note that the instructions request that we include this in the cover letter only and that the editorial office will update this information in the online submission form.

3. Please include captions for your Supporting Information files at the end of your manuscript, and update any in-text citations to match accordingly. Please see our Supporting Information guidelines for more information: <http://journals.plos.org/plosone/s/supporting-information>.

We have included captions for our Supporting Information files at the end of our manuscript and have updated all references to these files in the main text.

4. We are unable to open your Supporting Information file Data2_Typology_Cluster.sav. Please kindly revise as necessary and re-upload.

Apologies, we have transferred the data from this SPSS .sav file into a new Excel .xlsx file format to ensure that it can be opened.

5. Please review your reference list to ensure that it is complete and correct. If you have cited papers that have been retracted, please include the rationale for doing so in the manuscript text, or remove these references and replace them with relevant current references. Any changes to the reference list should be mentioned in the rebuttal letter that accompanies your revised manuscript. If you need to cite a retracted article, indicate the article’s retracted status in the References list and also include a citation and full reference for the retraction notice.

We have reviewed the reference list and can confirm it is complete and correct and that it does not include any papers which to our knowledge have been retracted.

**Comments to the Author**

1. Is the manuscript technically sound, and do the data support the conclusions?

The manuscript must describe a technically sound piece of scientific research with data that supports the conclusions. Experiments must have been conducted rigorously, with appropriate controls, replication, and sample sizes. The conclusions must be drawn appropriately based on the data presented.

Reviewer #1: Yes

Reviewer #2: Yes

2. Has the statistical analysis been performed appropriately and rigorously?

Reviewer #1: Yes

Reviewer #2: Yes

3. Have the authors made all data underlying the findings in their manuscript fully available?

The [PLOS Data policy](https://eur03.safelinks.protection.outlook.com/?url=http%3A%2F%2Fwww.plosone.org%2Fstatic%2Fpolicies.action%23sharing&data=05%7C01%7Cj.allan%40abdn.ac.uk%7C6055c768c26d4b4a856408db9371109d%7C8c2b19ad5f9c49d490773ec3cfc52b3f%7C0%7C0%7C638265886633825430%7CUnknown%7CTWFpbGZsb3d8eyJWIjoiMC4wLjAwMDAiLCJQIjoiV2luMzIiLCJBTiI6Ik1haWwiLCJXVCI6Mn0%3D%7C3000%7C%7C%7C&sdata=K6a1Qb8UCKU06JSPnG61Jsg0EBV%2FPaCKEsGDRVuwn8w%3D&reserved=0) requires authors to make all data underlying the findings described in their manuscript fully available without restriction, with rare exception (please refer to the Data Availability Statement in the manuscript PDF file). The data should be provided as part of the manuscript or its supporting information, or deposited to a public repository. For example, in addition to summary statistics, the data points behind means, medians and variance measures should be available. If there are restrictions on publicly sharing data—e.g. participant privacy or use of data from a third party—those must be specified.

Reviewer #1: Yes

Reviewer #2: Yes

4. Is the manuscript presented in an intelligible fashion and written in standard English?

PLOS ONE does not copyedit accepted manuscripts, so the language in submitted articles must be clear, correct, and unambiguous. Any typographical or grammatical errors should be corrected at revision, so please note any specific errors here.

Reviewer #1: Yes

Reviewer #2: Yes

5. Review Comments to the Author

Please use the space provided to explain your answers to the questions above. You may also include additional comments for the author, including concerns about dual publication, research ethics, or publication ethics. (Please upload your review as an attachment if it exceeds 20,000 characters)

Reviewer #1: I have carefully read the manuscript and my opinion is that the manuscript has a merit to be published in your reputable journal with some minor corrections. The manuscript is original, informative and readable. This study uses a systematic and structured expert consensus (Delphi) approach plus cluster-analysis to create a formal physical activity demand (PAD) typology which quantifies and then classifies the cognitive, physical, and social demands of common physical activity types. The introduction is well written, while materials and methods section is very well prepared and organized according to contemporary methodological rules. At the end, I have no amendments on results and discussion as well as conclusion part but I would recommend to the authors to exclude ethical approval subsection from the method section as it is technical information that should be placed in some additional section such as acknowledgement or something similar. Lastly, I would recommend you to accept this manuscript right after I confirm the authors revise it in the adequate manner.

Thank you for your positive feedback on this manuscript. As the PLOS ONE instructions for authors require that an ethics statement containing the name of approving institutional review board and a statement about whether participants gave written or oral informed consent is included in the Methods section of papers, we have not moved the statement as suggested.

Reviewer #2: The abstract does not have a proper structure. It is written as guidelines. Nothing about the sample..., the method of measurement...
Interesting study. The authors show an exceptional level of experience. I recommend that the study be published in its current form. Of course with Abstract corrections

Thank you for your positive feedback, and apologies for the omission of the abstract information highlighted. The abstract has been amended to include more information about the sample, methods and results, while continuing to adhere to the journal’s guidance on structure.

6. PLOS authors have the option to publish the peer review history of their article ([what does this mean?](https://eur03.safelinks.protection.outlook.com/?url=https%3A%2F%2Fjournals.plos.org%2Fplosone%2Fs%2Feditorial-and-peer-review-process%23loc-peer-review-history&data=05%7C01%7Cj.allan%40abdn.ac.uk%7C6055c768c26d4b4a856408db9371109d%7C8c2b19ad5f9c49d490773ec3cfc52b3f%7C0%7C0%7C638265886633825430%7CUnknown%7CTWFpbGZsb3d8eyJWIjoiMC4wLjAwMDAiLCJQIjoiV2luMzIiLCJBTiI6Ik1haWwiLCJXVCI6Mn0%3D%7C3000%7C%7C%7C&sdata=H8aFuNZqmiGEr95HOL%2BDufEXkYMWNmk%2BYnq8AkZY6Gc%3D&reserved=0)). If published, this will include your full peer review and any attached files.

If you choose “no”, your identity will remain anonymous but your review may still be made public.

**Do you want your identity to be public for this peer review?** For information about this choice, including consent withdrawal, please see our [Privacy Policy](https://eur03.safelinks.protection.outlook.com/?url=https%3A%2F%2Fwww.plos.org%2Fprivacy-policy&data=05%7C01%7Cj.allan%40abdn.ac.uk%7C6055c768c26d4b4a856408db9371109d%7C8c2b19ad5f9c49d490773ec3cfc52b3f%7C0%7C0%7C638265886633825430%7CUnknown%7CTWFpbGZsb3d8eyJWIjoiMC4wLjAwMDAiLCJQIjoiV2luMzIiLCJBTiI6Ik1haWwiLCJXVCI6Mn0%3D%7C3000%7C%7C%7C&sdata=uRhCc2AN%2FaM9OOcRxFkx2nNRysFk7OhB6wWuVRllOuQ%3D&reserved=0).

Reviewer #1: **Yes:** Stevo Popovic

Reviewer #2: No
